# Physician group practice in China's healthcare reform: a national survey on physicians' perspectives and implementation challenges

**DOI:** 10.3389/frhs.2025.1574388

**Published:** 2025-06-30

**Authors:** Rui Fan, Qing Meng, Long Zhang, Chao Liu, Li Han, Meng Liu, Leiyu Shi

**Affiliations:** ^1^ Department of Health Policy Management, Bloomberg School of Public Health, Johns Hopkins University, Baltimore, MD, United States; ^2^ IAE de Paris-Sorbonne Business School, University Paris 1 Pantheon Sorbonne, Paris, France; ^3^ Physician Group Department, Chinese Non-government Medical Institutions Association, Guangzhou, China; ^4^ Department of Critical Care Medicine, The First Medical Center of Chinese People’s Liberation Army General Hospital, Beijing, China; ^5^ Young and Middle-Aged Physicians Department, China Information Association of Traditional Chinese Medicine, Beijing, China

**Keywords:** physician group practice, healthcare reform, health policy, Chinese healthcare system, medical workforce management

## Abstract

**Background:**

The physician group (PG) model, while well-established in the United States, is a relatively recent healthcare delivery innovation in China. Despite rapid growth in PG registrations, comprehensive understanding of physicians’ perspectives remains limited.

**Objective:**

To investigate Chinese physicians’ perspectives and concerns regarding the PG model and identify factors influencing their support for its implementation.

**Methods:**

A cross-sectional online survey was conducted among 535 Chinese physicians between October-November 2024. The survey assessed participants’ views on PG advantages, concerns, and overall support. Data analysis included descriptive statistics and variance analyses to explore correlations between physicians’ characteristics and their perspectives.

**Results:**

Key facilitators for PG development included “New career direction outside the existing system” (75.1%) and “Improved income levels” (74.4%). Major concerns comprised “Policies restricting physician mobility” (69.7%) and “Lack of support from hospital managers” (57.8%). Overall support for PGs was moderate (3.710 ± 1.241). Administrative position holders showed significantly higher support than non-administrative staff (*p* = 0.004), and longer work experience correlated positively with support (*p* = 0.037). Hospital level and specialty area showed no significant influence on support levels.

**Conclusion:**

This first systematic study of Chinese physicians’ perspectives on PGs reveals moderate support driven by career development and income opportunities, while highlighting regulatory and institutional barriers. Results suggest the need for stable policies, enhanced income frameworks, and targeted support for early career physicians-who currently show less support than experienced physicians-to facilitate successful PG implementation in China.

## 1 Introduction

A physician group is a collective of healthcare professionals collaborating within a common practice, sharing resources to deliver patient care. This model, originating at the Mayo Clinic over a century ago (1), was officially introduced in China in 2014, with more than 1,000 groups registered by 2019 (2). Although the COVID-19 pandemic temporarily hindered growth, physician groups have since resumed activity in the post-pandemic period.

The national policy guideline “Healthy China 2030,” published in 2016, marked the first official mention of the physician group model in a national document, encouraging the exploration of this approach (3). Recent policies have increasingly supported physician mobility and independent practice. These include the implementation of tiered diagnosis systems and medical alliances that facilitate resource sharing between physician groups and hospitals, promoting the distribution of quality healthcare resources across different regions (4). Additionally, digital platforms have enabled the development of “mobile physician groups,” reducing information asymmetry between doctors and patients while enhancing physician brand recognition (5).

Research on physician groups in the United States indicates that group practices enhance physician quality of life and job satisfaction by facilitating knowledge transfer among members and reducing professional isolation. Patients in these group practices typically benefit from improved quality of care, better access to services, and higher satisfaction compared to those in solo practices (1, 6). These advantages of the physician group model are crucial for addressing specific challenges within the Chinese healthcare system.

The purpose of this study is to explore the perspectives and concerns of Chinese physicians regarding the physician group model, as well as to identify the key factors influencing these concerns. By gathering insights directly from healthcare providers, the research aims to highlight the barriers and facilitators associated with the implementation of physician groups in China. Understanding these perspectives is essential for developing effective strategies that support the successful integration of this model into the Chinese healthcare system (7). Ultimately, the study seeks to contribute to the ongoing dialogue about healthcare reform in China and to provide actionable recommendations that can enhance physician collaboration, improve patient outcomes, and foster a more resilient healthcare infrastructure.

## 2 Methods

### 2.1 Study design

The questionnaire was developed through a comprehensive literature review, and subsequently revised and expanded based on feedback from a group of 16 experienced pilot physicians who are knowledgeable or experts in this field. Their valuable feedback helped to enhance the questionnaire's content and structure, ensuring its relevance and accuracy in exploring the subject matter.

The inclusion criteria for participating physicians were as follows: (1) currently practicing in China; (2) holding a professional title such as resident, attending physician, associate chief, or chief; (3) possessing experience working in a physician group practice or having a solid understanding of the physician group practice model; and (4) being able to communicate effectively in Mandarin or Cantonese. To ensure that the sample consisted solely of qualified individuals, the exclusion criteria eliminated medical students, nurses, technicians, and those with a medical background who are not currently practicing as physicians. This approach was designed to focus on individuals who could provide meaningful insights and perspectives regarding the physician group model within the Chinese healthcare system.

The survey was distributed using Wenjuan Star, a widely used online questionnaire tool in China, in combination with WeChat, a popular social media platform, to facilitate effective data collection. Conducted from October 31st to November 22nd, 2024. The survey reached a broad audience across China, supported by collaborations with the Chinese Non-government Medical Institutions Association and the China Information Association of Traditional Chinese Medicine. These supports were essential in ensuring the survey was disseminated widely, thereby enhancing the diversity and representativeness of the responses.

The questionnaire is structured into five distinct sections. The first section gathers demographic information, including professional titles, years of experience, and geographical location. The second section assesses respondents' knowledge through a series of yes-or-no questions. The third section further explores practical experiences by employing a similar yes-or-no format. The fourth section utilizes a 5-point Likert scale to evaluate attitudes, allowing participants to indicate their levels of agreement or disagreement with various statements. Finally, the fifth section comprises multiple-choice questions aimed at identifying the factors that facilitate or hinder the implementation of Physician Groups. This comprehensive structure facilitates a thorough collection of data on key aspects related to Physician Groups, encompassing demographics, knowledge, practice experiences, attitudes, and influential factors.

The primary aim of this study is to explore physicians' attitudes toward Physician Groups (PG) and analyze the influencing factors. To achieve this, the research focuses on demographic analysis and the examination of various factors that may affect these attitudes. Additionally, the study will highlight several crucial questions related to attitudes, contributing to a comprehensive understanding of how different elements interplay in shaping physicians' perceptions of PG.

### 2.2 Ethical considerations

This study was reviewed by the Institutional Review Board Office of Johns Hopkins Bloomberg School of Public Health on January 9th, 2024. The IRB Determination notice (FWA #00000287) stated that the proposed activity does not qualify as human subjects research, as defined by DHHS regulations 45 CFR 46.102, and therefore does not require IRB oversight. The study involved collecting information from individuals about matters unrelated to themselves, without disclosing personal opinions and without exposing respondents to employment or other risks. Additionally, the study ensured participant anonymity and confidentiality by using questionnaires that did not collect names or emails, thus preventing participant tracking. Informed consent was obtained through the questionnaire, and participation was voluntary. The entire study was conducted without any financial burden to the participants.

### 2.3 Data analysis

The data were analyzed using descriptive statistics to summarize the characteristics of the sample population. Facilitators and barriers associated with physicians' choices to join physician groups (PGs) were ranked based on the frequency of responses. A differential analysis was performed to evaluate attitudes towards PGs across various demographic profiles, including years of experience, education, hospital management roles, hospital level, specialty, and geographic location. A significance level of *p* < 0.05 was considered indicative of statistical differences. Statistical analyses were conducted using SPSS v24.0 software. Furthermore, we examined the correlation between demographic variables and physicians' attitudes, suggesting avenues for future research to explore additional factors influencing perceptions of PG Model.

## 3 Results

### 3.1 Demographic profile

The study population consisted of 535 physicians affiliated with various medical institutions across China. In terms of education, the majority (46.4%) held a bachelor's degree or below, followed by master's degree holders (35.5%), and a smaller proportion (18.1%) possessing a doctorate or higher degree. The doctors' years of work experience ranged from less than five years to more than two decades, with a significant proportion (35.9%) having over 20 years of clinical practice. Their professional titles spanned from resident doctors (5.8%) to chief physicians (20.4%), with associate chief physicians (40%) being the most represented. Specializing in various medical fields, the majority (61.1%) were in surgery, followed by internal medicine (18.7%), traditional Chinese medicine (6.7%), and others (13.5%). Regarding hospital administrative positions, about two-thirds of the doctors did not hold administrative roles within their hospitals. In terms of hospital level, the study population was predominantly from tertiary hospitals with over 500 beds (61.9%), followed by secondary and specialty hospitals. The participants were also geographically diverse, with the eastern economic belt being the most represented (55.9%), followed by the central and western economic belts, as well as Hong Kong, Macau, and Taiwan (Table 1).

**Table 1:** Demographic profile (*N* = 535).

Characteristic	Subgroup	Frequency	Percent
Years of Work Experience	5 Years or Less	34	6.4
	6–10 Years	56	10.5
	11–15 Years	143	26.7
	16–20 Years	110	20.6
	Over 20 Years	192	35.9
	Total	535	100
Professional Title	Resident Doctor	31	5.8
	Attending Physician	181	33.8
	Associate Chief Physician	214	40
	Chief Physician	109	20.4
	Total	535	100
Hospital administrative position	Yes	204	38.1
	No	331	61.9
	Total	535	100
Hospital Level	Tertiary (More than 500 beds)	331	61.9
	Secondary and below	131	24.5
	Specialty Hospital	13	2.4
	Missing	60	11.2
Region	Northeast China (Heilongjiang, Jilin, Liaoning)	26	4.9
	Eastern Economic Belt (Beijing, Tianjin, Hebei, Shanghai, Jiangsu, Zhejiang, Fujian, Shandong, Guangdong, Hainan)	299	55.9
	Central Economic Belt (Shanxi, Anhui, Jiangxi, Henan, Hubei, Hunan)	74	13.8
	Western Economic Belt (Inner Mongolia, Guangxi, Chongqing, Sichuan, Guizhou, Yunnan, Tibet, Shaanxi, Gansu, Qinghai, Ningxia, Xinjiang)	131	24.5
	Hong Kong, Macau, and Taiwan	5	0.9
	Total	535	100

### 3.2 Facilitators of PG model

The analysis of facilitators for the development of physician groups (PGs) revealed several key drivers. The most frequently cited facilitator was the opportunity for a new career direction outside the existing system (75.1%), followed by improved income levels (74.4%) and guarantees for physician autonomy (69.5%). Transparent market earnings, reducing career risks (68.8%), and medical capabilities improvement (59.4%) were also prominent factors. Additionally, promoting the market flow of high-quality medical resources (56.4%) and improving patient satisfaction (54.2%) were highlighted. Enhancing physician job satisfaction (53.5%) and strengthening non-public healthcare systems (45.0%) were noted as further advantages (Table 2).

**Table 2:** Multi-choice results for facilitators of PG (*N* = 535).

Facilitators	Frequency	Percent
New career direction outside the existing system	402	75.10%
Improved income levels	398	74.40%
Guarantees for physician autonomy	372	69.50%
Transparent market earnings, reducing career risks	368	68.80%
medical capabilities improvement	318	59.40%
Promoting the market flow of high-quality medical resources	302	56.40%
Improving patient satisfaction	290	54.20%
Enhancing physician job satisfaction	286	53.50%
Enhancing the non-public healthcare systems	241	45.00%
Other advantages	18	3.40%

Supplementary analyses were conducted to discern potential variations in the top 3 facilitators based on physicians’ demographic and professional characteristics. Factors such as education, years of work experience, professional title, specialty, administrative roles, and hospital level were evaluated to assess their influence on the perceived significance of these facilitators. These refined analyses aimed to uncover distinct patterns and subgroup differences, facilitating a deeper understanding of how diverse physician profiles align with the development of PGs (Table 3).

**Table 3:** Expanded analysis of top facilitators.

Characteristic	Subgroup	Total	New career direction outside the system (75.1%)				Improved income levels 74.4%)				Guarantees for physician autonomy (69.5%)			
			*n*	%	*χ* ^2^	*P*	*n*	%	*χ* ^2^	*P*	*n*	%	*χ* ^2^	*P*
Education	Bachelor's and below	248	179	72.2	2.329	0.312	177	71.4	2.223	0.329	183	73.8	3.957	0.138
	Master's Degree	190	149	78.4			146	76.8			125	65.8		
	Doctorate or Above	97	74	76.3			75	77.3			64	66.0		
Years of Work Experience	5 Years or Less	34	26	76.5	2.37	0.668	23	67.6	11.33	0.023*	20	58.8	5.529	0.237
	6–10 Years	56	44	78.6			47	83.9			40	71.4		
	11–15 Years	143	110	76.9			106	74.1			93	65.0		
	16–20 Years	110	85	77.3			91	82.7			76	69.1		
	Over 20 Years	192	137	71.4			131	68.2			143	74.5		
Professional Title	Resident Doctor	31	25	80.6	4.509	0.212	17	54.8	7.828	0.050*	20	64.5	4.812	0.186
	Attending Physician	181	129	71.3			138	76.2			116	64.1		
	Associate Chief	214	159	74.3			157	73.4			156	72.9		
	Chief Physician	109	89	81.7			86	78.9			80	73.4		
Specialty	Internal Medicine	100	72	72.0	3.676	0.299	75	75.0	3.695	0.296	60	60.0	5.75	0.124
	Surgery	327	252	77.1			249	76.1			236	72.2		
	Traditional Chinese	36	29	80.6			27	75.0			24	66.7		
	other	72	49	68.1			47	65.3			52	72.2		
Hospital administrative position	Yes	204	149	73.0	0.779	0.377	145	71.1	1.901	0.168	148	72.5	1.416	0.234
	No	331	253	76.4			253	76.4			224	67.7		
Hospital Level	Tertiary (500+ beds)	331	259	78.2	4.214	0.122	260	78.5	6.326	0.042*	239	72.2	2.204	0.332
	Secondary and below	131	91	69.5			89	67.9			86	65.6		
	Specialty Hospital	13	9	69.2			11	84.6			10	76.9		

* *p* < 0.05.

#### 3.2.1 New career direction outside the existing system (75.1%)

A new career direction was the most frequently cited facilitator, with 75.1% of respondents identifying this advantage. Although there were no significant differences in responses based on education (*p* = 0.312), years of work experience (*p* = 0.668), or other major factors (Table 3), several trends emerge:

Chief physicians (81.7%) rated this facilitator higher compared to attending physicians (71.3%) and associate chief physicians (74.3%). This may suggest that physicians at the pinnacle of their professional journey increasingly seek alternatives to traditional institutional roles.

Surgeons were more likely to emphasize career opportunities (77.1%) compared to internal medicine doctors (72%). This difference, while not statistically significant (*p* = 0.299), may reflect a higher alignment of surgical fields with entrepreneurial or independent practice opportunities often presented in physician groups (PGs).

Importantly, tertiary hospital physicians, who are typically affiliated with large institutions (78.2%), also saw PGs as a means to step outside the limitations of these systems, compared to those in secondary hospitals (69.5%, *p* = 0.122). This highlights PGs' potential as a response to the rigidity of traditional practice models, offering flexibility and diversification.

These findings suggest that fostering messaging around “career transformation” and emphasizing independence in marketing or policy campaigns could resonate strongly with certain physician subgroups, especially senior physicians or those engaged in high-demand specialties like surgery.

#### 3.2.2 Improved income levels (74.4%)

Income improvement was the second most popular facilitator, significantly influenced by various demographic factors:

Physicians with 6–10 years of work experience rated this factor highest, with 83.9% selecting it as a facilitator (*p* = 0.023). In contrast, older physicians (over 20 years of experience) showed lower support (68.2%). Younger and mid-career doctors may view PGs as a means to address financial inequalities earlier in their careers compared to their senior colleagues, who are closer to retirement and may prioritize other factors like stability.

Physicians in tertiary hospitals were significantly more likely to view income improvement as a key facilitator (78.5%) compared to those in secondary hospitals (67.9%, *p* = 0.042). This reflects the restrictive salary structures of public hospitals, where tertiary-level institutions often impose tighter financial frameworks despite demanding workloads.

Interestingly, the professional title variable showed borderline significance (*p* = 0.050), with chief physicians (78.9%) and attending physicians (76.2%) slightly more likely to emphasize this factor compared to resident doctors (54.8%). This indicates that targeting salary disparities and creating incentive-based payment frameworks could be an effective strategy for PG implementation. Policies allowing transparent revenue-sharing systems and market-driven earnings may attract wider support from younger and mid-career physicians, particularly in tertiary hospitals.

#### 3.2.3 Guarantees for physician autonomy (69.5%)

Guaranteeing physician autonomy was the third most prominent facilitator, viewed as critical across different demographics. While no statistically significant differences emerged across key factors, several notable trends highlight the importance of autonomy in driving PG adoption:

Chief physicians (73.4%) and associate chief physicians (72.9%) were more inclined to rate autonomy as critical, compared to attending physicians (64.1%). This aligns with the higher expectation among experienced physicians to exercise greater decision-making power over clinical and administrative processes.

Physicians in tertiary hospitals (72.2%) rated autonomy higher than those in secondary hospitals (65.6%); meanwhile, differences across specialties suggested a similar trend, with internal medicine physicians rating it lower (60%) compared to surgeons (72.2%) and practitioners of Traditional Chinese Medicine (66.7%). This suggests that physicians in niche or procedural specialties may perceive more constraints in the current healthcare system compared to others.

### 3.3 Barriers about PG model

The analysis of barriers to the development of physician groups (PGs) revealed several critical challenges cited by respondents. The most frequently reported barrier was policies restricting physician mobility, identified by 69.7% of participants, reflecting substantial concerns about regulatory constraints within the current healthcare system. This was followed by the lack of support from hospital managers (57.8%) and unstable and unsustainable income (57.2%), both of which highlight organizational and financial difficulties faced by physicians considering the transition to PGs. Additionally, over half of the respondents identified challenges such as limited career development opportunities (53.8%) and insufficient academic resources (53.6%), demonstrating dissatisfaction with institutional barriers hindering professional growth and access to knowledge. Structural and operational obstacles, including a lack of a clear and sustainable business model (50.8%) and limited brand value to attract patients (41.3%), were also noted. Other notable concerns included the shortage of hospitals providing qualified supporting services (40.0%) and limited support from health insurance (39.6%), both of which reflect systemic deficiencies in infrastructure and financial backing. Finally, approximately one-third of respondents highlighted insufficient business operation capabilities (34.2%) as a significant barrier, while only 2.6% selected “Other Challenges,” indicating the primary concerns were well represented (Table 4).

**Table 4:** Multi-choice results for barriers of PG (*N* = 535).

Barriers	Frequency	Percent
Policies restricting physician mobility	373	69.70%
Lack of support from hospital managers	309	57.80%
Unstable and unsustainable income	306	57.20%
Limited career development opportunities	288	53.80%
Insufficient academic resources	287	53.60%
Lack of a clear and sustainable business model	272	50.80%
Lack of brand value to attract patients	221	41.30%
Shortage of hospitals providing qualified supporting services	214	40.00%
Limited support from health insurance	212	39.60%
Insufficient business operation capabilities)	183	34.20%
Other Challenges	14	2.60%

Supplementary analyses were performed to explore potential variations in the top barriers—policies restricting physician mobility, lack of support from hospital managers, and unstable and unsustainable income—based on physicians' demographic and professional characteristics (Table 5).

**Table 5:** Expanded analysis of top 3 barriers.

Characteristic	Subgroup	Total	Policies restricting physician mobility (69.7%)				Lack of support from hospital managers (57.8%)				Unstable and unsustainable income (57.2%)			
			*n*	%	*χ* ^2^	*P*	*n*	%	*χ* ^2^	*P*	*n*	%	*χ* ^2^	*P*
Education	Bachelor's and below	248	174	70.2	3.928	0.14	147	59.3	0.535	0.765	137	55.2	1.68	0.432
	Master's Degree	190	139	73.2			106	55.8			108	56.8		
	Doctorate or Above	97	60	61.9			56	57.7			61	62.9		
Years of Work Experience	5 Years or Less	34	25	73.5	1.817	0.769	19	55.9	1.432	0.839	18	52.9	0.968	0.915
	6–10 Years	56	37	66.1			34	60.7			33	58.9		
	11–15 Years	143	102	71.3			77	53.8			85	59.4		
	16–20 Years	110	80	72.7			65	59.1			64	58.2		
	Over 20 Years	192	129	67.2			114	59.4			106	55.2		
Professional Title	Resident Doctor	31	21	67.7	0.881	0.83	14	45.2	3.445	0.328	14	45.2	2.656	0.448
	Attending Physician	181	122	67.4			104	57.5			103	56.9		
	Associate Chief Physician	214	153	71.5			122	57.0			122	57.0		
	Chief Physician	109	77	70.6			69	63.3			67	61.5		
Specialty	Internal Medicine	100	66	66.0	7.362	0.061	57	57.0	6.638	0.084	53	53.0	1.391	0.708
	Surgery	327	237	72.5			197	60.2			191	58.4		
	Traditional Chinese	36	28	77.8			23	63.9			19	52.8		
	other	72	42	58.3			32	44.4			43	59.7		
Hospital administrative position	Yes	204	137	67.2	1.026	0.311	115	56.4	0.259	0.611	109	53.4	1.909	0.167
	No	331	236	71.3			194	58.6			197	59.5		
Hospital Level	Tertiary (500 + beds)	331	239	72.2	0.585	0.746	207	62.5	8.202	0.017*	196	59.2	2.376	0.305
	Secondary and below	131	90	68.7			65	49.6			69	52.7		
	Specialty Hospital	13	9	69.2			10	76.9			9	69.2		

* *p* < 0.05.

#### 3.3.1 Policies restricting physician mobility (69.7%)

“Policies restricting physician mobility” was the most frequently cited barrier, chosen by 69.7% of respondents. This reflects a widespread concern regarding the regulatory constraints that limit physicians' ability to transition between institutions or participate in physician groups (PGs). While the chi-square analysis reveals no statistically significant differences across most demographic and professional variables, some trends are noteworthy:

Respondents with a master's degree were slightly more likely to identify this barrier (73.2%) compared to those with a bachelor's or below (70.2%) and those with a doctorate or above (61.9%, *p* = 0.14). Physicians specializing in Traditional Chinese Medicine (TCM) reported the highest proportion (77.8%) identifying physician mobility restrictions as a barrier, followed by surgical specialists (72.5%) and internal medicine practitioners (66.0%, *p* = 0.061). The relatively higher percentages in TCM and surgery may suggest these professionals desire greater flexibility for collaborative opportunities or practice expansion.

#### 3.3.2 Lack of support from hospital managers (57.8%)

The second most frequently cited challenge was “lack of support from hospital managers,” chosen by 57.8% of respondents. Unlike the first barrier, this factor showed a statistically significant relationship with hospital level (*p* = 0.017). Physicians working in tertiary hospitals with over 500 beds were significantly more likely to cite this barrier (62.5%) than those in secondary and below hospitals (49.6%), with a very high proportion seen in specialty hospitals (76.9%). Tertiary hospitals tend to have more rigid hierarchies and managerial systems, which may hinder the flexibility needed for PG participation. While not statistically significant (*p* = 0.084), TCM practitioners (63.9%) and surgical specialists (60.2%) were slightly more likely than internal medicine specialists (57.0%) and those in “other” specialties (44.4%) to perceive managerial resistance.

#### 3.3.3 Unstable and unsustainable income (57.2%)

The third most frequently noted barrier was “unstable and unsustainable income,” identified by 57.2% of respondents. While this challenge did not present statistically significant differences across most demographics. Interestingly, respondents with a doctorate or higher were more likely to report this barrier (62.9%) than their counterparts with a master's degree (56.8%) or a bachelor's degree or below (55.2%, *p* = 0.432). This suggests that physicians with higher educational qualifications may experience heightened expectations for financial reliability that are unmet in the PG model. Chief physicians (61.5%) and associate chief physicians (57.0%) expressed greater concerns about income instability compared to attending (56.9%) and resident doctors (45.2%, *p* = 0.448).

### 3.4 Physicians' perception on “I support PG to develop in China”

The overall support for the development of physician groups (PGs) in China was positive, with a mean score of 3.710 ± 1.241 among all respondents (*n* = 535), where a score of 3 indicates neutrality. Further analysis revealed significant variations across several demographic and professional characteristics (Table 6).

**Table 6:** Physicians’ characters to statement “I support PG to develop in China”.

Characteristic	Subgroup	*n* = 535	Mean ± sd	*F*	*P*
Total		535	3.710 ± 1.241		
Education	Bachelor's and below	248	3.810 ± 1.201	1.971	0.14
	Master's Degree	190	3.574 ± 1.265		
	Doctorate or Above	97	3.722 ± 1.281		
Years of Work Experience	5 Years or Less	34	3.382 ± 1.256	2.571	0.037*
	6–10 Years	56	3.571 ± 1.219		
	11–15 Years	143	3.587 ± 1.147		
	16–20 Years	110	3.673 ± 1.342		
	Over 20 Years	192	3.922 ± 1.232		
Professional Title	Resident Doctor	31	3.226 ± 1.383	2.546	0.055
	Attending Physician	181	3.624 ± 1.165		
	Associate Chief Physician	214	3.790 ± 1.248		
	Chief Physician	109	3.835 ± 1.280		
Hospital administrative position	Yes	204	3.907 ± 1.214	2.897	0.004**
	No	331	3.589 ± 1.243		
Region	Northeast China	26	3.654 ± 1.384	3.211	0.013*
	Eastern Economic Belt	299	3.689 ± 1.280		
	Central Economic Belt	74	4.149 ± 0.822		
	Western Economic Belt	131	3.542 ± 1.273		
	Hong Kong, Etc.	5	3.200 ± 1.304		

* *p* < 0.05.

** *p* < 0.01.

Years of Work Experience showed a significant relationship with support levels (*F* = 2.571, *p* = 0.037). Notably, physicians with over 20 years of experience demonstrated the strongest support (3.922 ± 1.232), while those with 5 years or less experience showed the lowest support (3.382 ± 1.256). This suggests that more experienced physicians may better recognize the potential benefits of PGs based on their extensive clinical experience.

Hospital Administrative Position emerged as another significant factor (*F* = 2.897, *p* = 0.004). Physicians holding administrative positions showed significantly higher support (3.907 ± 1.214) compared to those without administrative roles (3.589 ± 1.243), indicating that healthcare leaders may better understand the strategic value of PGs in addressing systemic challenges.

Regional differences were also significant (*F* = 3.211, *p* = 0.013), with physicians in the Central Economic Belt showing the strongest support (4.149 ± 0.822), notably higher than their counterparts in other regions, particularly the Western Economic Belt (3.542 ± 1.273) and Hong Kong, Macau, and Taiwan (3.200 ± 1.304).

While not statistically significant, there were observable trends across other variables. Professional titles showed a marginal effect (*F* = 2.546, *p* = 0.055), with chief physicians (3.835 ± 1.280) and associate chief physicians (3.790 ± 1.248) expressing stronger support compared to resident doctors (3.226 ± 1.383). Similarly, specialty hospitals demonstrated the highest mean support (4.077 ± 1.320) among hospital levels, though this difference was not statistically significant (*F* = 0.972, *p* = 0.379).

## 4 Discussion

### 4.1 Key findings

#### 4.1.1 Overall support with demographic variations

The study shows that Chinese physicians exhibit a generally positive attitude toward the development of physician groups (PGs), with an average support score of 3.710 ± 1.241. This highlights the openness of medical professionals to adopt innovative care models to address systemic issues in China's healthcare system (8, 9) (e.g., uneven resource distribution, lack of autonomy). Previous studies support the conclusion that healthcare restructuring efforts like PGs often receive strong backing from medical professionals willing to improve efficiency and patient outcomes (10). This positive attitude is essential for advancing policy frameworks around PG adoption in China.

Physicians with over 20 years of experience expressed remarkable support (3.922 ± 1.232, *p* = 0.037), aligning with findings that experience shapes confidence in independent models like PGs. Senior physicians likely recognize the inefficiencies of traditional hospital systems and value PGs' potential to enhance autonomy and income (11). Additionally, physicians in administrative roles demonstrated significantly higher support (3.907 ± 1.214, *p* = 0.004), as they are more familiar with organizational decision-making and the potential operational efficiencies of PGs (12, 13). This indicates that institutional leaders could act as advocates for PG implementation.

Regional differences highlight a higher readiness for PG adoption in the Central Economic Belt, which showed the strongest support (4.149 ± 0.822, *p* = 0.013). These regions often experience a unique balance of resource availability, greater medical competition, and economic advancements, contributing to stronger acceptance of innovative models. In contrast, lower support in the Western Economic Belt (3.542 ± 1.273) correlates with systemic resource and infrastructure limitations. Tailored strategies recognizing these regional disparities should direct implementation efforts toward regions with higher readiness, while addressing barriers in under-resourced areas (14, 15).

#### 4.1.2 Career development and financial incentives as primary facilitators

The opportunity for new career directions emerged as the most cited facilitator (75.1%), highlighting physicians' growing desire for alternatives to traditional institutional roles. Many physicians, especially senior specialists, view physician groups (PGs) as a solution to rigid institutional hierarchies and limited professional growth opportunities. This finding is consistent with the Diffusion of Innovations theory, which posits that adoption of new organizational models such as PGs is influenced by perceived advantages—including autonomy and professional advancement—over existing systems. This aligns with broader global trends, where healthcare professionals increasingly prioritize entrepreneurial ventures and independent practice models for career satisfaction (1, 16). By enabling greater flexibility, PGs offer physicians a platform to redefine their professional track while meeting the evolving demands of healthcare services.

Improved income levels (74.4%) were identified as the second most important facilitator, indicating strong financial expectations from joining PGs. Physicians in mid-career stages (6–10 years) and those in tertiary hospitals, who often experience restrictive salary structures, were particularly motivated by income improvement. Transparent revenue-sharing systems and opportunities for market-driven earnings make PGs attractive for physicians seeking to bridge wage disparities. These financial incentives represent a critical driver, especially for healthcare providers who are overburdened yet undercompensated within traditional employment frameworks (17, 18).

#### 4.1.3 Systemic and institutional barriers

Policy restrictions on physician mobility emerged as the most significant barrier, with 69.7% of participants identifying this issue. Regulatory constraints hinder physicians from transitioning between institutions or joining physician groups (PGs), limiting opportunities for professional growth or alternative employment. Simplifying inter-institutional approval system and promoting multi-site licensing could help alleviate this challenge and enable greater mobility within the healthcare system (8, 19).

Lacking institutional support from hospital managers was reported by 57.8% of respondents, with the issue being more acute in tertiary hospitals (62.5%, *p* = 0.017). Large, hierarchical institutions often display resistance to external frameworks like PGs, as managers prioritize maintaining control over talent networks. Addressing these barriers requires targeted engagement with hospital leaders, promoting options like co-management models that align PG goals with institutional objectives (20–22).

Concerns about unstable and unsustainable income were highlighted by 57.2% of participants. Notably, this issue was more prominent among highly educated physicians (e.g., those with doctoral degrees, 62.9%). Higher expectations for financial security, derived from their advanced qualifications, may explain greater sensitivity to income risks compared with less educated peers. Transparent revenue-sharing mechanisms and government-backed financial incentives for PG participants could address these concerns while ensuring economic stability (23). Furthermore, tailoring financial structures for PGs to meet the needs of senior and highly educated physicians could enhance engagement (24, 25).

### 4.2 Implications and suggestions

#### 4.2.1 Policy reform priorities

Policy reform to enhance physician mobility is critical for the successful development of PGs. Current regulatory constraints limit physicians' ability to explore opportunities outside traditional systems. To address this, comprehensive reforms should introduce flexible mobility structures that facilitate inter-institutional transitions (26). For example, designing transferable contracts or creating inter-regional licensing systems can enable physicians to engage in collaborative, multi-site practices without bureaucratic hurdles (27). Enhanced flexibility will ensure optimal utilization of medical expertise across regions while reducing professional waste (28–30).

International experience, particularly from the United States, offers valuable insights. In the US, physicians often hold state-specific licenses, but mechanisms such as the Interstate Medical Licensure Compact (IMLC) have been developed to facilitate multi-state practice and physician mobility (31). This compact allows eligible physicians to obtain expedited licensure in multiple participating states, thereby reducing administrative barriers and enabling greater workforce flexibility—especially important for large physician groups and telemedicine practices (32, 33). Similarly, models from other low- and middle-income countries (LMICs), such as India's Apollo Group, demonstrate how adaptable policy environments and innovative contracting can support the growth of physician groups despite resource constraints (34). Comparisons with these international models highlight both the potential and the challenges of implementing cross-regional practice frameworks in China's unique healthcare context.

Clear legal protections for physicians participating in PGs are paramount to overcome risks associated with independent practice. Without robust legal frameworks, physicians face uncertainties regarding income stability, liability, and intellectual property. Policymakers should establish legal safeguards addressing these issues to create a supportive ecosystem for PG members.

Reforms must be tailored to address regional disparities in healthcare infrastructure and willingness to adopt PGs. For example, to advance physician group (PG) integration and operational effectiveness, pilot programs for multi-site licensing reforms can be initiated in regions such as the Central Economic Belt, which already demonstrates an active physician group landscape and physician mobility. These pilots would test streamlined licensing processes that allow qualified physicians to practice across multiple institutions and regions, reducing administrative barriers and broadening access to high-quality clinical expertise. Conversely, less prepared areas such as the Western Economic Belt may need capacity-building initiatives to strengthen institutional cooperation before implementing large-scale reforms. These targeted approaches can bridge gaps, ensuring equitable development and expanding healthcare access nationwide (35).

#### 4.2.2 Institutional support mechanisms

To foster the integration of Physician Groups (PGs) into the broader healthcare ecosystem, it is crucial to establish collaborative models between PGs and hospitals. Many hospitals, particularly tertiary institutions, demonstrate resistance to PGs due to the perceived threat of losing skilled medical professionals. Addressing this requires co-management models that align the objectives of both parties. For example, hospitals can collaborate with PGs through shared service platforms, resource pooling, and coordinated patient care pathways. Such collaborations improve patient outcomes while reducing inter-institutional conflicts, paving the way for successful PG adoption (36, 37).

Transparent revenue-sharing systems are essential for encouraging physician participation in PGs and lightening financial concerns. A fair and standardized revenue-sharing framework can provide clarity on how profits are distributed among participating physicians and affiliated hospitals. These systems should also ensure that financial incentives align with performance metrics, fostering a culture of accountability and trust while improving physician satisfaction and operational sustainability (38, 39).

Creating structured professional development pathways within PGs can further enhance their appeal. Physicians often identify limited career growth opportunities in traditional hospital systems. As such, PGs should offer tailored career trajectories, including specialized training programs, leadership development courses, and research initiatives (40). By fostering an environment that prioritizes educational advancement and skill-building, PGs can attract top-tier talent and empower their members to achieve long-term professional success (41, 42). These development pathways could also address regional and institutional disparities by nurturing more skilled physicians in underserved areas.

### 4.3 Limitations

This study has several limitations that deserve attention. First, the sample distribution reveals notable regional imbalances, with overrepresentation from the Eastern and Central Economic Belts, and disproportionate representation from certain areas such as the Central Economic Belt. These disparities may limit the generalizability of the findings to all regions of China. While we did not apply statistical weighting to adjust for these differences, we explicitly acknowledge this as a limitation and recommend the use of stratified or weighted sampling designs in future research to improve representativeness.

Second, our reliance on self-reported data introduces potential bias, as respondents may overstate or misrepresent their knowledge, attitudes, or practices regarding physician group (PG) development. The anonymous, voluntary nature of the survey may have resulted in nonresponse bias, with individuals more interested in or supportive of PGs being more likely to participate. This could further skew the results and limit their applicability to the broader population of healthcare professionals.

Third, the cross-sectional design of the study restricts our ability to draw causal inferences about the factors influencing support for PGs. Longitudinal research would be beneficial to track changes in knowledge, attitudes, and practices over time.

Finally, our analysis was limited to descriptive statistics, as sample constraints precluded multivariate regression. Consequently, we could not fully assess the influence of potential confounders such as hospital funding models, regional GDP, and institutional policy variations. These unmeasured factors may have impacted our results. We recommend that future studies use multivariate analyses and more comprehensive data collection to better clarify the interplay of institutional, economic, and regional variables, as well as to enhance generalizability through more balanced samples and longitudinal approaches.

## 5 Conclusion

The findings highlight the widespread support among Chinese physicians for the development of PGs, with senior practitioners and those in administrative roles exhibiting the strongest advocacy. Facilitators such as career development, financial incentives, and autonomy emerge as key motivators, while barriers including restrictive mobility policies and income stability concerns underscore systemic challenges. To succeed, PGs need policy reforms enabling flexibility, institutional support through collaborative models, and tailored financial and professional growth systems. Addressing these challenges holistically can maximize the potential of PGs, enabling them to transform healthcare delivery and improve patient outcomes throughout China.

## Institutional review board statement

This study was reviewed by the Institutional Review Board Office of Johns Hopkins Bloomberg School of Public Health on January 9th, 2024. The IRB Determination notice (FWA #00000287) stated that the proposed activity does not qualify as human subjects research, as defined by DHHS regulations 45 CFR 46.102, and therefore does not require IRB oversight.

## Data Availability

The raw data supporting the conclusions of this article will be made available by the authors, without undue reservation.
